# Financial Network Connectedness and Systemic Risk During the COVID-19 Pandemic

**DOI:** 10.1007/s10690-021-09340-w

**Published:** 2021-06-14

**Authors:** Mike K. P. So, Lupe S. H. Chan, Amanda M. Y. Chu

**Affiliations:** 1 Department of Information Systems, Business Statistics and Operations Management, The Hong Kong University of Science and Technology, Clear Water Bay, Hong Kong; 2 Department of Social Sciences, The Education University of Hong Kong, Tai Po, Hong Kong

**Keywords:** Financial contagion, Granger causality, Network density, Pandemic network, Risk analytics

## Abstract

The COVID-19 pandemic causes a huge number of infections. The outbreak of COVID-19 has not only caused substantial healthcare impacts, but also affected the world economy and financial markets. In this paper, we study the effect of the COVID-19 pandemic on financial market connectedness and systemic risk. Specifically, we test dynamically whether the network density of pandemic networks constructed by the number of COVID-19 confirmed cases is a leading indicator of the financial network density and portfolio risk. Using rolling-window Granger-causality tests, we find strong evidence that the pandemic network density leads the financial network density and portfolio risk from February to April 2020. The findings suggest that the COVID-19 pandemic may exert significant impact on the systemic risk in financial markets.

## Introduction

The coronavirus disease 2019 (COVID-19) pandemic has led to more than 150 million confirmed cases and more than 3 million deaths as of 30 April 2021 (WHO, 2021). Many researchers focused on the modeling of financial returns during COVID-19. For example, Maleki et al. (2020) proposed a time series model with asymmetric errors for forecasting the worldwide death rate of COVID-19. Guliyev (2020) introduced a spatial panel data approach to study the spatial effects of COVID-19. For analyzing the relationship between COVID-19 and financial risk, especially the financial contagion effect, Shehzad et al. (2020) employed an asymmetric power GARCH model for analyzing the effects of COVID-19 on financial markets worldwide. As one may expect, financial markets can become very volatile as a result of COVID-19 pandemic. Shehzad et al. (2020) showed that the COVID-19 pandemic has significant effects on several financial markets in different countries. Broadie et al. (2015) proposed a regression-based nested Monte Carlo simulation method for financial risk estimation. However, relatively few studies investigated the effects of COVID-19 on systemic risk in financial markets. In this paper, we fill such research gap by investigating the impact of the COVID-19 pandemic on financial network connectedness and systemic risk.

We develop an approach to study the financial contagion (The New York Times, 2020) effect from the COVID-19 pandemic using network analysis, a set of integrated techniques used to depict relations among nodes (Chiesi, 2001). Billio et al. (2012) proposed several econometric measures of network connectedness for analyzing the inter-relationship of the financial risk in finance and insurance sectors. So et al. (2021a) studied the impact of COVID-19 on financial market connectedness. In this paper, we consider two sets of nodes: the daily returns of market indices and the daily changes in the square root of the number of confirmed cases of COVID-19 in different markets or countries (So et al., 2020). Using the methods in Chu et al. (2020a, 2020b), Tiwari et al. (2021), and So et al. (2021b) who proposed the use of network density to detect early signals of the COVID-19 pandemic, we test any lead-lag relationships between the pandemic network and the financial network by testing the Granger causality between the pandemic network density and the financial network density. Three key findings from this paper are noteworthy. First, we observe a time lag of a few days between the first peak of the pandemic network density and the peak of the financial network density, signifying possible lead-lag relationship between the financial network and the pandemic network. Second, the tests for the pandemic network to Granger-cause the financial network are significant from March to April 2020, meaning that the pandemic network density significantly affects the financial network density and the systemic risk from March to April 2020. Finally, there is high proportion of random portfolios being Granger-caused by the pandemic network density from April to May 2020, indicating that portfolio risk can be much affected by the pandemic from April to May 2020.

The rest of the paper is as follows. Section 2 describes how we construct dynamic financial and pandemic networks and presents statistical methods for investigating financial contagion from the COVID-19 pandemic. Section 3 provides financial network properties during COVID-19 and studies the lead-lag relationship between financial and pandemic networks. Section 4 is the discussion.

## Materials and Methods

### Financial Market Connectedness and Systemic Risk

#### Dynamic Financial Networks

Systemic risk refers to the breakdown of a financial system. The breakdown can be caused by a series of contagion activities or events. There are various methods in assessing systemic risk. One approach in quantifying systemic risk is by building financial networks to capture possible contagion in financial systems (Demange, 2016; Elliott et al., 2014; Gai & Kapadia, 2010; Glasserman & Young, 2016). In the literature, high systemic risk can be attributable to high connectedness in financial securities where breakdown of some financial institutions or markets can be transmitted to, or can “infect”, other markets, causing widespread breakdown of financial systems and leading to increase in systemic risk. In this paper, we assess the systemic risk of international financial markets over time by constructing financial networks dynamically. A dynamic network is a sequence of graphs indexed in time order and denoted by $G_t=(V_t,E_t)$, where $V_t$ is a set of vertices (or nodes) at time *t* and $E_t$ is a set of edges at time *t*, where $t=1, ..., T$. In our case, the edges are undirected, i.e., only connectedness is considered. Each node in the financial networks represents a market and that in the pandemic networks represents a country.

To examine the connectedness of two market indices at time *t*, we calculate the partial correlations of the market log returns with MSCI World Index log returns and MSCI Emerging Markets Index log returns as covariates. If the partial correlation of the log returns between two indices is greater than a threshold value, $r_F$, the two indices are regarded as connected and we refer it as a connection in a financial network. Once we construct the network, we then examine network characteristics to understand the impact of the COVID-19 pandemic on financial market connectedness. Mathematically, suppose we have data from $N+K$ markets (the log returns of *K* indices are used to be the control covariates) for *T* days. Let $P_{it}$ be the adjusted closing price of the *i*-th index in day *t*, for $i=1,\ldots ,N+K$ and $t=1,\ldots ,T$. Indices $1,\ldots ,N$ are used to build the dynamic financial networks and indices $N+1,\ldots ,N+K$ are used as control covariates for partial correlation calculation. Below are the steps to construct the financial network in day *t*. In our case, $K=2$.

1. Let $R_{it}$ be the log return of the *i*-th index in day *t*, which is calculated as $R_{it}=\log P_{it}-\log P_{i(t-1)}$, for $t=2,\ldots ,T$.
2. Let $${\mathbf {R}}_{t}^{(N)}=\begin{bmatrix}R_{1t}&R_{2t}&\ldots&R_{Nt}\end{bmatrix}^T$$ be the vector of returns for the first *N* indices and $${\mathbf {R}}_{t}^{(M)}=\begin{bmatrix}R_{(N+1)t}&R_{(N+2)t}&\ldots&R_{(N+K)t}\end{bmatrix}^T$$ be the log returns of the indices used to be the control covariates. Also use $\varSigma _{NN,t}$ and $\varSigma _{MM,t}$ to denote respectively their covariance matrices. Let $w>1$ be the window size for the covariance estimation. An estimator for ${\varvec{\mu }}_{\varvec{t}}$, the population mean of ${\mathbf {R}}_t^{(N)}$, is ${\hat{{\varvec{\mu }}}}_{{\varvec{t}}}(w)=\frac{1}{w}\sum _{s=t-w+1}^t {\mathbf {R}}_s^{(N)},$ and an estimator for $\varSigma _{NN,t}$, the covariance matrix for ${\mathbf {R}}_t^{(N)}$ is
  $$\begin{aligned} {\hat{\varSigma }}_{NN,t}(w)=\frac{1}{w-1}\sum _{s=t-w+1}^t ({\mathbf {R}}_s^{(N)}-{\hat{{\varvec{\mu }}}}_{{\varvec{t}}}(w))({\mathbf {R}}_s^{(N)}-{\hat{{\varvec{\mu }}}}_{{\varvec{t}}}(w))^T, \end{aligned}$$
  for $t=w,\ldots ,T$. The formula holds similarly for the estimation of other covariance matrices.
3. Define the variance-covariance matrix
  $$\begin{aligned} \mathrm {Var}\left( \begin{bmatrix} {\mathbf {R}}_t^{(N)}\\ {\mathbf {R}}_t^{(M)}\end{bmatrix} \right) = \begin{bmatrix} \varSigma _{NN,t} &{} \varSigma _{NM,t}\\ \varSigma _{MN,t} &{} \varSigma _{MM,t} \end{bmatrix}, \end{aligned}$$
  where the (*i*, *j*)-th entry of $\varSigma _{NM,t}$ is $\mathrm {Cov}(R_{it}^{(N)},R_{jt}^{(M)})$ and $\varSigma _{MN,t}=\varSigma _{NM,t}^T$ .
  Then, the partial correlation matrix is $R_{N|M,t} = (\mathrm {diag}(\varSigma _{N|M,t}))^{-1/2}\varSigma _{N|M,t}(\mathrm {diag}(\varSigma _{N|M,t}))^{-1/2},$
  where $$\varSigma _{N|M,t} = \mathrm {Var}({\mathbf {R}}_t^{(N)}|{\mathbf {R}}_t^{(M)})=\varSigma _{NN,t}-\varSigma _{NM,t}\varSigma _{MM,t}^{-1}\varSigma _{MN,t},$$ for $$t=w,\ldots ,T$$.
4. Define a connection between market *i* and market *j* if the partial correlation between two markets is greater than $r_F$.

#### Network Statistics of the Dynamic Financial Networks

Network statistics are attributes from a network that summarize the properties or characteristics of a network. For dynamic networks, we have a set of network statistics at a particular time point and so we can obtain time series for the network statistics. Network density is defined as the ratio of the number of connected edges to the number of all possible edges, i.e., $D_t^{(F)} = \frac{2|E_t|}{|V_t|(|V_t|-1)},$ where $|E_t|$ and $|V_t|$ denote the number of edges and vertices of the financial network at time *t*, respectively. Clustering coefficient is a measure of the degree of tendency of nodes clustering together. The global clustering coefficient is defined as the ratio of the number of closed triplets to the total number of triplets (open and closed) (Newman, 2018).

Network centrality can be used to examine the strength of the connections in a network. Let $A_t=[A_{ij,t}]$ be the adjacency matrix of the dynamic financial network in day *t*, also let $${\mathbf {c}}_t=\begin{bmatrix}c_{1,t}&c_{2,t}&\ldots&c_{N,t}\end{bmatrix}^T$$ be the vector of network centralities of the financial network of all markets in day *t*, with $c_{i,t}>0$ for all $i=1,2,\ldots ,N$. The network centrality of market *i* is defined as $c_{i,t} = \frac{1}{\lambda }\sum _{j=1}^N A_{ij,t}c_{j,t},$ which can be obtained by calculating the principal eigenvector of $A_t$ by solving the equation $A_t{\mathbf {c}}_t = \lambda {\mathbf {c}}_t$, since only the entries in the principal eigenvector are guaranteed to be non-negative.

### Financial Contagion from COVID-19 Pandemic

A main objective of this paper is to investigate the impact of the COVID-19 pandemic on the contagion in international financial markets. Following Chu et al. (2020a, 2020b), So et al. (2021b), and Tiwari et al. (2021), we obtain the network densities for the pandemic networks according to the steps below. Let $X_{it}$ be the number of confirmed cases of country *i* in day *t*. Calculate $Y_{it}=\sqrt{X_{it}}-\sqrt{X_{i(t-1)}}$, where $X_{it}$ is the newly confirmed cases of a country *i* in day *t*. Find the correlation of $Y_{it}$ and $Y_{jt}$ in day *t* using data of $w=14$ days, including the past 13 days and day *t*. Construct the COVID-19 pandemic network in day *t* by linking country *i* and country *j* when the correlation between two countries is larger than 0.5. We denote the number of (nonoverlapping) connections of the pandemic network in day *t* by $|E_t^{(P)}|$. Calculate the pandemic network density in day *t* as $D_{t}^{(P)}=\frac{2|E_t^{(P)}|}{C_t(C_t-1)},$ where $C_t$ is the number of countries included in the pandemic network in day *t*.

#### Granger Causality Test

After obtaining the network densities for the financial networks and the pandemic networks, we calculate the log returns of the network densities for the financial networks and the pandemic networks as $d_t^{(F)}$ and $d_t^{(P)}$. Specifically, we calculate $d_t^{(F)} = \log D_t^{(F)} - \log D_{t-1}^{(F)}~ \text { and } ~d_t^{(P)}=\log D_t^{(P)} - \log D_{t-1}^{(P)}.$ Then, we conduct Granger causality tests to investigate if the pandemic networks “Granger-cause” the financial networks.

Granger causality test is a joint hypothesis test for the causality between two time series. Statistically, we want to test

1 $$\begin{aligned} \begin{aligned} H_0:&~d_{t}^{(F)} =\beta _0+\sum _{i=1}^p \beta _i d_{t-i}^{(F)}+\varepsilon _t^{(F)}, \\ H_1 :&~d_{t}^{(F)} =\beta _0+\sum _{i=1}^p \beta _i d_{t-i}^{(F)}+\sum _{j=1}^q \alpha _i d_{t-j}^{(P)}+\varepsilon _t, \end{aligned} \end{aligned}$$

or equivalently, $H_0: ~\alpha _1=\alpha _2=\ldots =\alpha _q=0$ versus $H_1: ~\text {at least one of the }\alpha _i's\text { is non-zero}$ using the data from day $t-w_{GC}+1$ to day *t*, where $w_{GC}$ is the moving window size of the Granger causality test. This is a F-test (or an ANOVA), with the test statistic

$$\begin{aligned} F=\frac{\left( Res.S.S.\big |_{H_0}-Res.S.S.\big |_{H_1}\right) /q}{Res.S.S.\big |_{H_1}/(n-p-q-1)}\sim F_{q,n-p-q-1}, \end{aligned}$$

where *Res*.*S*.*S*. is the residual sum of squares of the regression in (1). We use the test to examine whether we have sufficient evidence to support the hypothesis that changes in the network density of the pandemic networks Granger-cause changes in the network density of the financial networks.

#### Risk Scores

We are also interested in how COVID-19 pandemic affects the performances of global investments. Suppose there are $N_{sim}$ simulated cases. Let $${\mathbf {w}}_i=\begin{bmatrix}w_{i1}&w_{i2}&\ldots&w_{iN}\end{bmatrix}^T$$ be the portfolio allocation vector of simulated case *i* with $\sum _{j=1}^N w_{ij}=1$. A measure to the performance of the portfolio allocation is the risk score, defined as

2 $$\begin{aligned} S_{it}=\sqrt{{\mathbf {w}}_i^TA_t{\mathbf {w}}_i}, \end{aligned}$$

where $A_t$ is the adjacency matrix of the financial network in day *t*.

A challenge for us is to obtain the real portfolio allocation data. Therefore, we conducted a simulation study as depicted below to evaluate the risk scores during the COVID-19 pandemic. We simulate $N_{sim}$ cases for the portfolio allocation, namely ${\mathbf {w}}_1,~{\mathbf {w}}_2,~\ldots ,~{\mathbf {w}}_{N_{sim}}$. A choice is to choose

3 $$\begin{aligned} {\mathbf {w}}_i \sim \text {Dirichlet}(\varvec{\alpha }), \end{aligned}$$

with a uniform choice of concentration parameter $${\varvec{\alpha }}=\underbrace{\begin{bmatrix}1&1&\ldots&1\end{bmatrix}^T}_{N\text { terms}}$$.

We then calculate all $S_{it}$, the risk scores in day *t* for each simulated case *i*. For the moving window size of $w_{GC}$, we conduct a Granger-Causality test in each day *t*, for $t=w_{GC},\ldots ,T$. Then, we can obtain empirical distributions of the *p*-values in each day *t*, as well as a time series of the proportion of significant case (at the significance level $\alpha$). We are interested in the period with high significant proportions.

## Empirical Results

### Financial Network Properties During COVID-19

**Table 1:** A list of the 47 stock markets, grouped by region and location, in this study

Region	Location	Short name	Full name
America	US	CCMP	NASDAQ Composite Index
	Brazil	IBOV	Bovespa Index
	US	INDU	Dow Jones Industrial Average Index
	Mexico	MEXBOL	S&P/BMV IPC Index
	US	RTY	Russell 2000 Index
	Canada	SPTSX	S&P/TSX Composite Index
	US	SPX	S&P 500 Index
	US	VIX	CBOE Volatility Index
Asia	Australia	AS51	S&P/ASX 200 Index
	China	DJSH	Dow Jones Shanghai Index
	Vietnam	HNX30	Hanoi Stock Exchange 30 Index
	China	HSI	Hang Seng Index
	Indonesia	JCI	Jakarta Stock Exchange Composite Index
	Korea	KOSPI	Korea Composite Stock Price Index
	India	NIFTY	NIFTY 50 Index
	Japan	NKY	Nikkei 225 Index
	New Zealand	NZDOW	Dow Jones New Zealand Index
	New Zealand	NZSE50FG	NZX 50 Index
	Philippines	PCOMP	PSE Composite Index
	India	SENSEX	BSE Sensex 30 Index
	Thailand	SET	SET Index
	China	SHCOMP	Shanghai Composite Index
	China	SICOM	SZSE Component Index
	Singapore	STI	FTSE Straits Times Singapore Index
	Taiwan	TWSE	Taiwan Weighted Index
	China	XIN9I	FTSE China A50 Index
Eastern	Pakistan	KSE100	KSE 100 Index
Mediter-ranean	Saudi Arabia	SASEIDX	Tadawul All Share Index
Europe	Netherlands	AEX	Amsterdam Exchange Index
	Austria	ATX	Austrian Traded Index
	France	BEL20	BEL 20 Index
	Hungry	BUX	Budapest SE Index
	France	CAC	CAC 40 Index
	German	DAX	DAX Index
	Italy	FTSEMIB	FTSE Milano Indice di Borsa Index
	Spain	IBEX	Índice Bursatil Espan̄ol 35 Index
	Russia	IMOEX	MOEX Russia Index
	Denmark	OMXC25	OMX Copenhagen 25 Index
	Sweden	OMXS30B	OMX Stockholm 30 Index
	Portugal	PSI20	Portuguese Stock Index 20 Index
	Russia	RTSI	Russia Trading System Index
	Switzerland	SMI	Swiss Market Index
	German	SX5E	EURO STOXX 50 Index
	Israel	TA-35	Tel Aviv 35 Index
	UK	UKX	Financial Times Stock Exchange 100 Index
	Poland	WIG20	Warszawski Indeks Giełdowy 20 Index
	Turkey	XU100	Borsa Istanbul 100 Index

We collect from Bloomberg the data of $N=47$ financial market indices, the MSCI world index and the MSCI emerging markets index, that is, $K=2$, taken as world market factors to control common market effects. A list of the 47 stock markets can be found in Table 1. The time coverage is 12 December 2019 to 29 May 2020 ($T=122$ trading days), which includes a few months before and after the COVID-19 pandemic being declared by the World Health Organization (WHO) on 11 March 2020. Moving partial correlation matrices on each trading day are then calculated with a window size of $w=40$ trading days. Effectively, we have the moving partial correlations from early February 2020 to late May 2020 since we use 40 trading days’ data to calculate the first moving partial correlation matrix. We construct dynamic financial dynamic networks, with the network link between two financial markets on a particular day being set up when their partial correlation is greater than $r_F=0.5$. As suggested by So et al. (2020), analyzing the COVID-19 pandemic network connectedness among regions can help visualize and estimate pandemic risk. In this paper, we show that analyzing the pandemic networks can also help estimate financial risk and the possible impact of the COVID-19 pandemic on the financial network evolution. From the relationship between the dynamic pandemic and financial networks, we test and quantify any contagion effect from the COVID-19 pandemic to the financial markets. Before performing statistical test on any contagion effect, we start from visualizing the dynamic financial networks to learn some insights.

#### Visualizing Dynamic Financial Networks

**Fig. 1:**
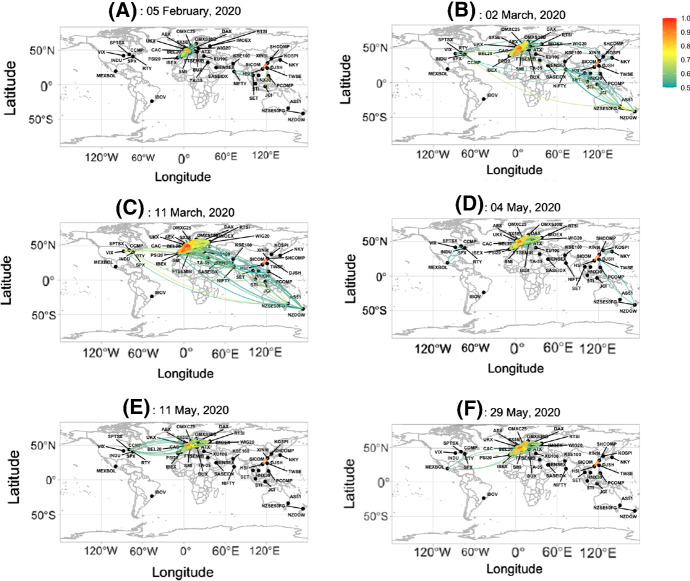
Network maps on the selected six days with the color indicating the values of the partial correlations

**Fig. 2:**
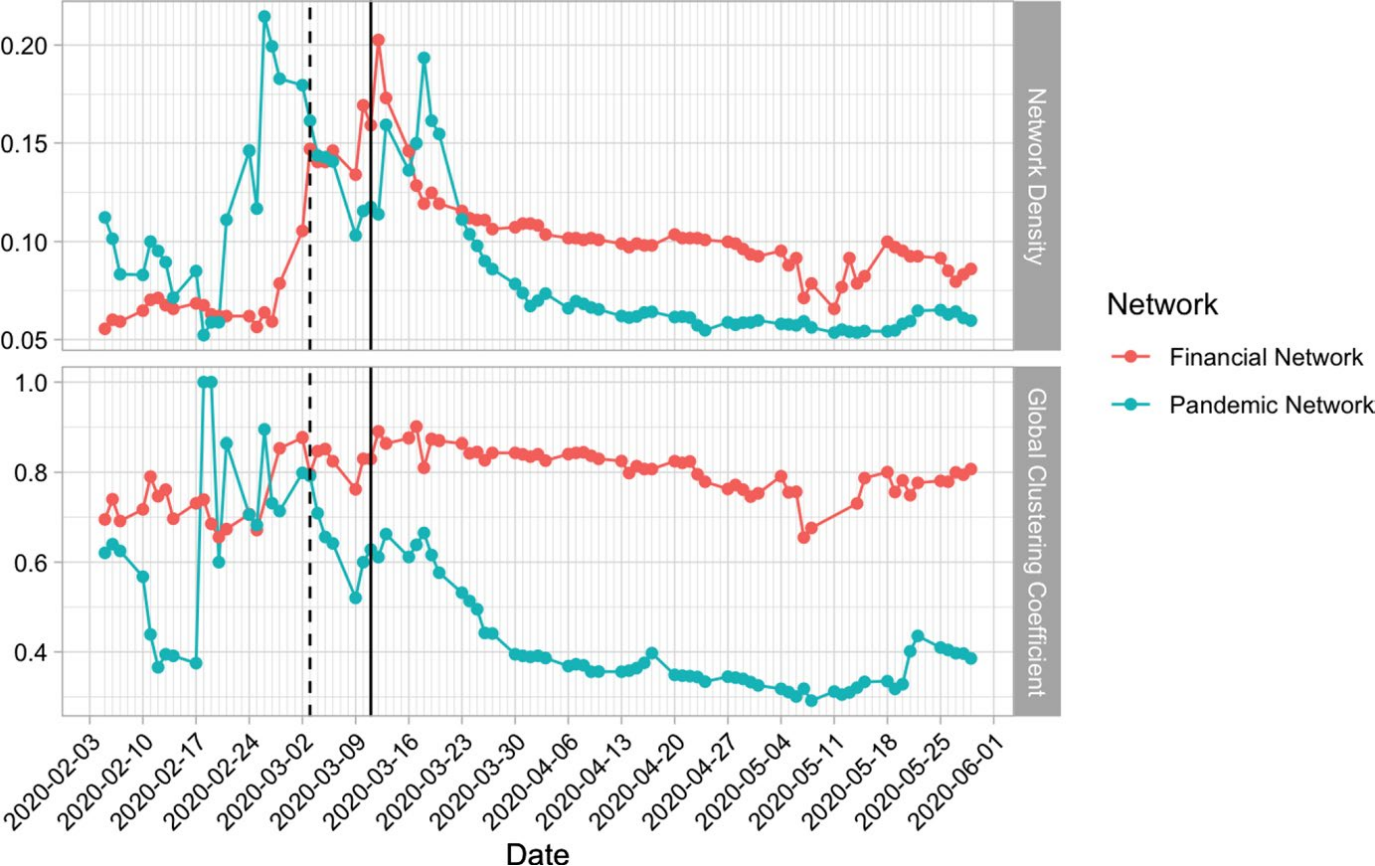
Time series plots of the pandemic network density and clustering coefficient (blue solid lines) and the financial network density and clustering coefficient (red solid lines)

As in the visualization of pandemic risk by So et al. (2020), possible financial risk can also be visualized based on the degree of connectedness among different stock markets in dynamic financial networks. Figure 1 presents graphically the financial networks constructed using the moving partial correlations. We select 6 days to illustrate how the financial market connectedness evolves over time as the COVID-19 pandemic. The six days are (A) 5 February 2020, (B) 2 March 2020, (C) the WHO’s declaration of the COVID-19 pandemic on 11 March 2020, (D) 4 May 2020, (E) 11 May 2020, and (F) 29 May 2020. We plot the networks in Fig. 1 with the connections represented by arcs with colors showing the magnitudes of the partial correlations. We give the description of the six selected days as follows:

(A) 5 February 2020, China reported over 10,000 confirmed cases, while most of the countries had less than 100 confirmed cases (Wikipedia, 2020). We take this day as our reference day for the pre-pandemic period.
(B) 2 March 2020, one week before 11 March. Both financial and pandemic networks became slightly more connected.
(C) 11 March 2020, the day WHO declaring COVID-19 as a global pandemic. The financial network’s connectedness was at a very high level.
(D) 4 May 2020. The market had already become less connected after pandemic fear.
(E) 11 May 2020. The day financial networks dropped sharply, which may be related to the news *World leaders pledge $8 billion to fight COVID-19 but U.S. steers clear* (Emmott & Guarascio, 2020) and *Catch up: Here are the latest coronavirus headlines you might have missed* (Regan et al., 2020).
(F) 29 May 2020. The global clustering coefficient of the pandemic networks went up again (Fig. 2). It was a sign of recurrence of the COVID-19 pandemic.

In Fig. 1A, there are not many links in the financial network. In early March shown in Fig. 1B, the financial markets are obviously more connected. The financial network shows very high connectedness on 11 March (Fig. 1C), when WHO declared COVID-19 as a global pandemic, indicating high systemic risk in the financial markets. The network map on 11 March shows dense connections inter-regionally that the financial contagion from the pandemic is quite highly evident. The network diagrams exhibit another high connectedness on 11 May 2020 in Fig. 1E. After that, the systemic risk seems to go down a bit as reflected in lower connectedness in Fig. 1F. The lower systemic risk may be attributed to some relief of the pandemic (Regan et al., 2020).

#### Financial and Pandemic Network Statistics

Figure 2 shows the time series plots of the network density and clustering coefficient of the financial networks (red solid lines) and the network density and clustering coefficient of the pandemic networks (blue solid lines) from Chu et al. (2020a). Note that the thick grid lines in the time series plots represent Monday and the thin grid lines in between represent other weekdays. In Fig. 2, the network density of the pandemic networks started to increase in mid February 2020 and reached a peak above 0.2 on 26 February 2020, two weeks before WHO declaring COVID-19 as a global pandemic (So et al., 2021b; Chu et al., 2020a). After the first spike of the pandemic network, the financial network started to rise but the rate of increase is smaller than that in the pandemic network. The first spike of the pandemic network seems to be an early signal for the contagion of pandemic risk to the financial markets. The financial network density reached the maximum of around 0.2 on 12 March 2020. To investigate any causal relationship between the financial and pandemic networks, we conduct the Granger-causality tests using the changes in the network density of the two networks in the next section.

The second spike of the pandemic network occurred on 18 March 2020. Unlike the first spike, the financial network did not respond too much to this increase in connectedness in the pandemic network. It may be a sign that the financial markets were adapting to the pandemic. The pandemic network density tended to decrease after the second spike. This decrease may be due to the tightening of the distancing measures in different countries in early March, including nationwide lockdowns (Deutsche Welle, 2020), reducing the spread of the pandemic. With the WHO’s announcement on 11 March 2020, various anti-pandemic measures were started to be set up by governments from different countries (Deutsche Welle, 2020). The stock markets hence started to recover themselves from the financial contagion. It can been seen from the decline of the financial network densities, or by comparing the network map on 11 March 2020 with the network map on 4 May 2020 in Fig. 1 that the inter-regional connections obviously reduced. The financial network densities were declining slowly until early May. Then, a sudden drop of the financial network density was observed in Fig. 2 on around 11 May 2020, which may be related to the news published on May 4, 2020, *World leaders pledge $8 billion for coronavirus treatments and vaccines* (Emmott & Guarascio, 2020).

We also present time series plots of the global clustering coefficients for both pandemic and financial networks in Fig. 2. The clustering coefficient of the pandemic networks rose up to 1.0 in mid February 2020, when the clustering coefficient of the financial networks exhibited an increasing trend from around 0.7. The financial clustering coefficient stayed at a high level of between 0.8 and 0.9 most of time in March 2020 and started declining after March 2020. Both the financial network’s and pandemic network’s clustering coefficients dropped to low values in April. However in May 2020, the two clustering coefficients stopped dropping, showing a sign of getting more severe in the pandemic. Many countries have eased lockdown restrictions in May 2020 (Pharmaceutical Technology, 2020), which may let our guard down, allowing the COVID-19 pandemic to swoop in again. We may carefully observe the pattern of the networks in the future in order to keep track of the pandemic and financial risks.

#### Network Centality of the Financial Networks during the COVID-19 Pandemic

**Fig. 3:**
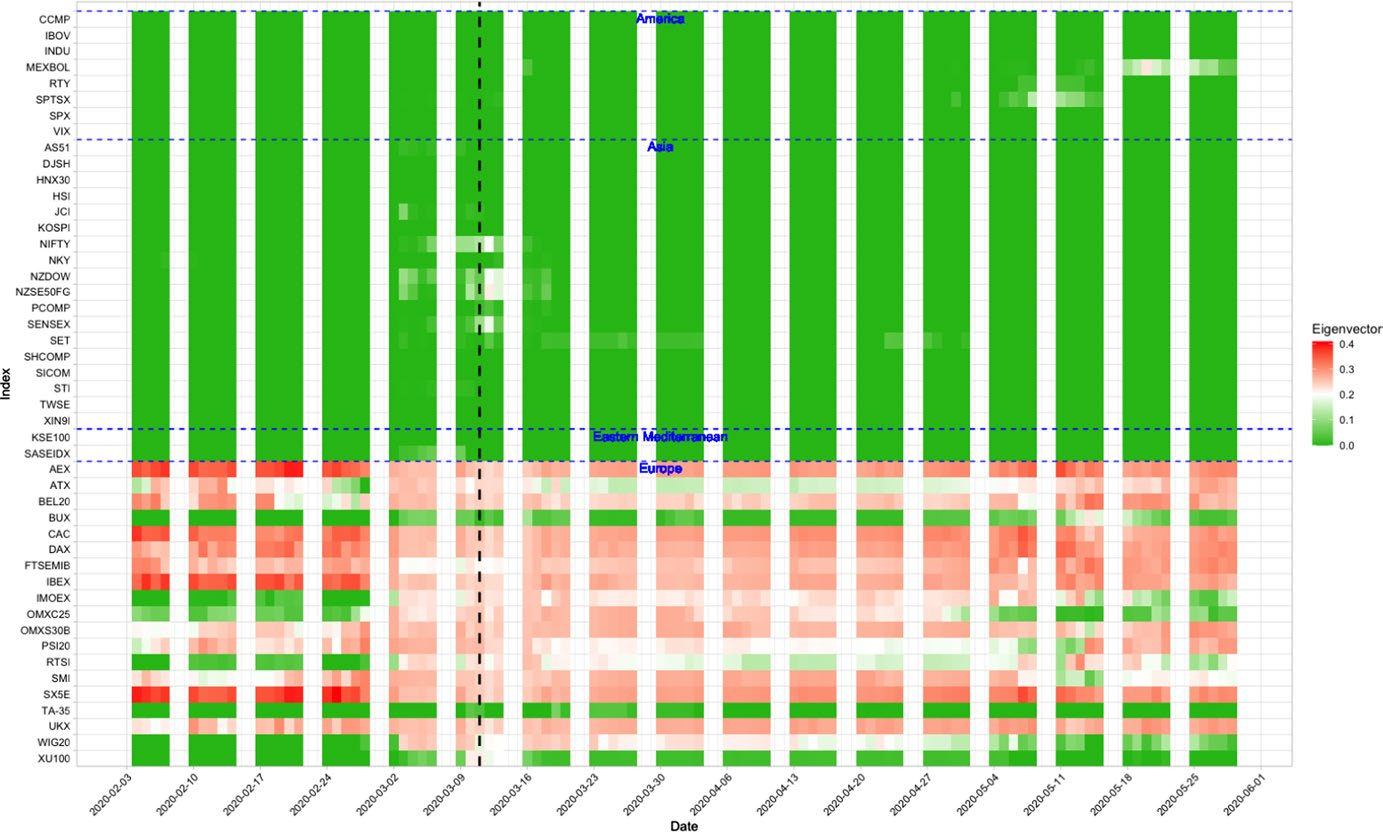
Heatmap of the network centrality of the financial markets. The black dashed line represents the 11 March, the day WHO declaring COVID-19 as a global pandemic

To measure the connectedness of a financial market, we calculate the network centrality of all markets, taking the influence of other markets into account. Figure 3 displays the heatmap of the network centrality of all 47 market indices from February to May 2020. In the heatmap, we divide the markets into four regions: America, Asia, Eastern Mediterranean and Europe based on the WHO’s classification of countries. We can see that the network centralities of many European indices are relatively high throughout the period of investigation from February to May 2020. From the heatmap of the network centrality in Fig. 3, the indices in Europe showed high centrality in February 2020. Some markets in Asia became more connected around the day of WHO’s announcement on 11 March 2020 (marked with vertical black dashed line), including NIFTY and SENSEX from India, and NZDOW and NZSE50FG from New Zealand. The changes in the centrality in the Indian and New Zealand markets may be partly attributed to the fact that the number of confirmed cases in India and New Zealand increased exponentially starting from the beginning of March 2020 (Wikipedia, 2021a, b). The network centrality of many markets in Europe, such as those in France, Germany, and United Kingdom, were getting smaller after the WHO’s announcement. The centrality for the European markets started to heat up again on around 11 May 2020, which is consistent with the news mentioned in Emmott & Guarascio (2020) and Regan et al. (2020). Markets in America including SPTSX from Canada and MEXBOL from Mexico were also slightly heating up in early May 2020 and late May 2020, respectively. Canada announced that they started to loosen some of their lockdown restrictions on 4 May 2020, and Mexico government allowed the state to reopen its businesses from 16 May 2020. These news may be related to the heat up of the SPTSX and MEXGOL. The heat map could also be used to predict financial risks. Early signs have been shown in the heat map right before the markets heat up in most of the cases. For example, on 11 May 2020, the network centrality for MEXBOL in Mexico started to rise. It was an early sign of the financial contagion in the week of 18 May 2020.

### Lead–Lag Relationship Between Pandemic and Financial Networks

**Fig. 4:**
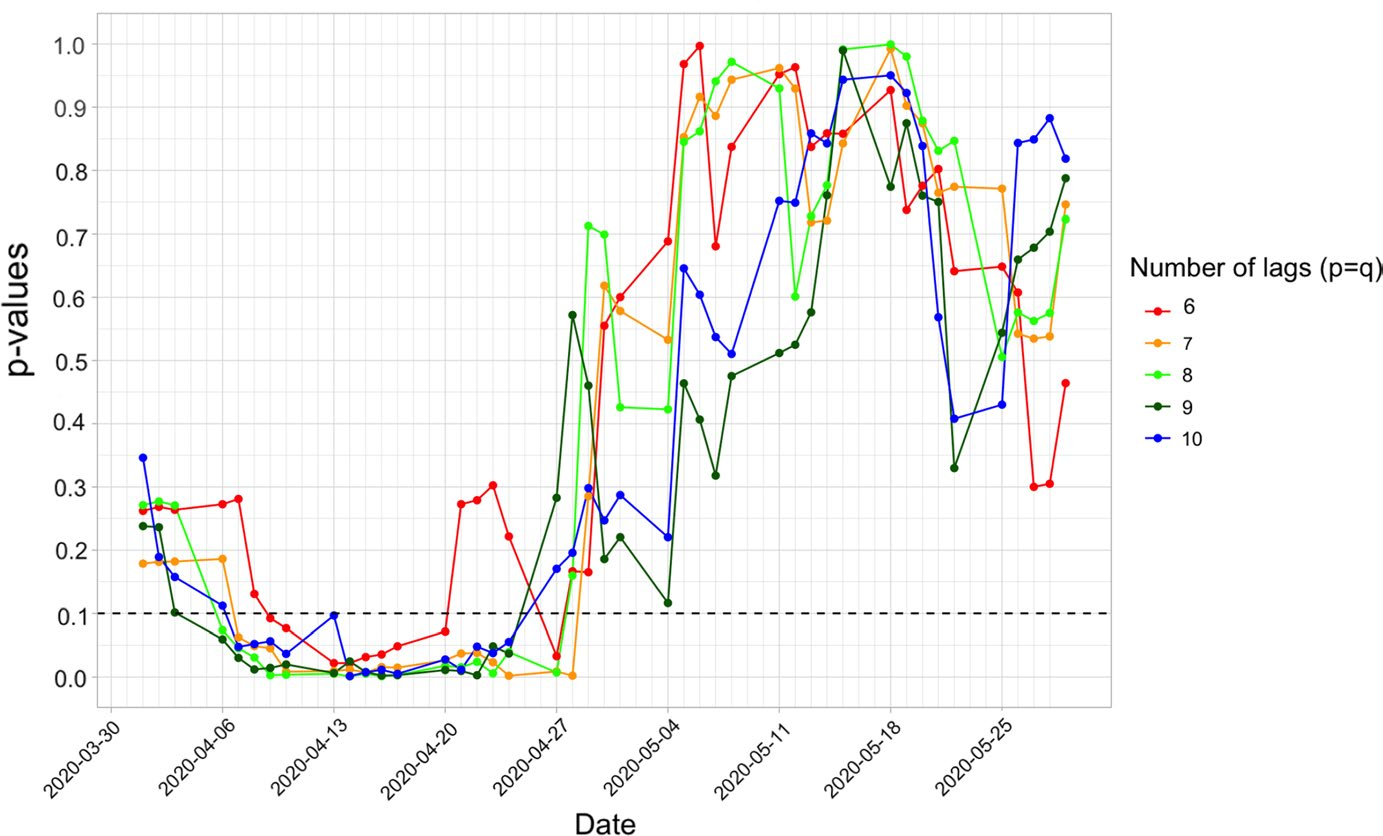
*P*-values of the moving-window Granger causality tests. The black dashed line represents $\alpha =0.1$, the critical values of the p-values for the Granger causality tests

#### Granger-causality Between Pandemic and Financial Networks

In Fig. 2, we observe that the first peak of the pandemic network density appeared in late February 2020, earlier than the peak of the financial network density. This finding indicates that the pandemic network connectedness may lead the financial network connectedness. To test whether the pandemic network density is a leading indicator of the financial network density, we test whether $d_t^{(P)}$ *Granger-causes* $d_t^{(F)}$, i.e., the pandemic networks contain information that help predict the financial networks. To account for the possible changing causality patterns in the pandemic and financial network density time series, we perform the Granger causality test in (1) using a moving-window mechanism, with the observations in the window, $t-w_{GC}+1$ to *t* to perform the test. We set the window size, $w_{GC}$, to 40 trading days and *t* is from 1 April 2020 to 29 May 2020. In other words, we perform the Granger causality test every trading day from the window [22 Feb 2020, 1 April 2020] to the window [20 April 2020, 29 May 2020] with 40-day observations in each window. We also choose the number of lags, $p=q=6,7,\ldots ,10$ in (1), for the sensitivity analysis of the test results.

Figure 4 shows the *p*-values of the test using different *p*’s and *q*’s, where the *x*-axis gives the end dates of the windows. The test results with different *p* and *q* are generally consistent. If we use the critical value of 0.1 for the Granger causality tests, the test results with end dates from 6 April 2020 to 24 April 2020 (network density data from 11 February 2020 to 24 April 2020 are involved in the tests) are significant for most *p*’s and *q*’s. In other words, we have sufficient evidence that the pandemic networks contain information that help predict the financial networks from 11 February 2020 to 24 April 2020, well-covering the time period when the COVID-19 was not that severe in February 2020 and when WHO declared COVID-19 as the pandemic on 11 March 2020. Therefore, the evidence on the assertion that the pandemic network density leads the financial network density is strong in February to April 2020. The above findings also imply the contagion from the COVID-19 pandemic to financial markets, causing substantial increase in systemic risk in financial markets. As indicated in Fig. 2, the pandemic network seems to be less influential to the financial network after early May 2020. The insignificant test results observed in Fig. 4 after early May align with the less influential effect.

**Fig. 5:**
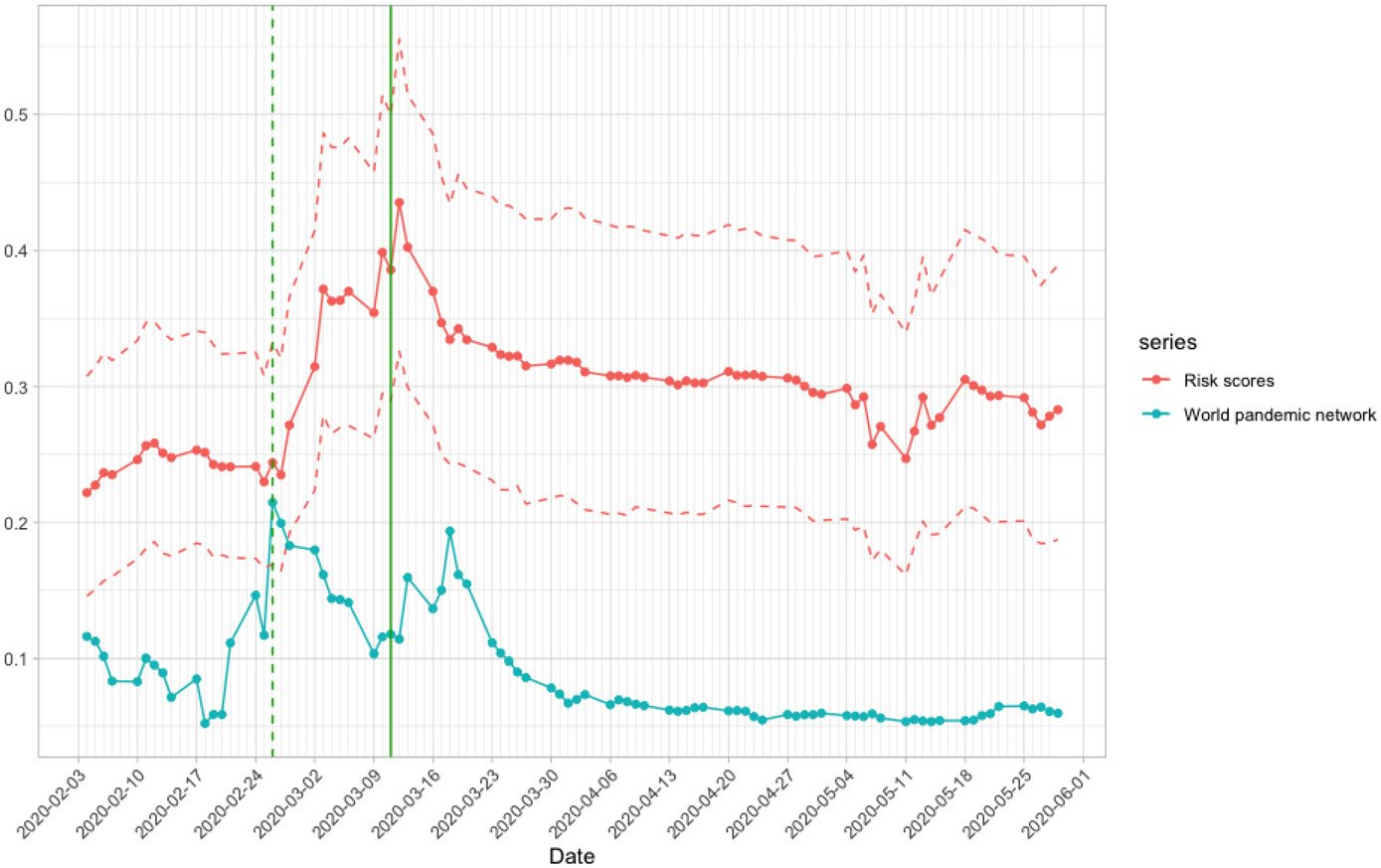
Time series plot for the average risk scores (red solid line). The red dashed lines are respectively the 2.5-th percentile and 97.5-th percentile of the simulated risk scores. The world pandemic network density (blue solid line) is also plotted. The green dashed line represents 26 February 2020, the peak of the world pandemic network density; the green solid line represents 11 March 2020, the day WHO declaring COVID-19 as a global pandemic

#### Does COVID-19 Granger-Cause Portfolio Risk?

To study the impact of the COVID-19 pandemic on portfolio risk, we simulate $N_{sim}$=1,000 random portfolios for investigation. Denote the random portfolios by ${\mathbf {w}}_1,~{\mathbf {w}}_2,~\ldots ,{\mathbf {w}}_{N_{sim}}$, where ${\mathbf {w}}_i$’s are independently simulated from a Dirichlet distribution in (3). Define the risk score for the *i*-th simulated portfolio in day *t* using Eq 2, i.e. $S_{it}=\sqrt{{\mathbf {w}}_i^TA_t {\mathbf {w}}_i}$. This risk score is based on the adjacency matrices, $A_t$, meaning that this score characterizes the portfolio risk due to the financial market connectedness. Figure 5 shows the time series plot of the average risk scores (red solid lines), the 2.5-th and 97.5-th percentiles (red dashed lines), and the world pandemic network density. We observe that the first spike of the world pandemic network density (on 26 February 2020, marked with a vertical dashed green line) occurred before that of the average risk scores (right after 11 March 2020, the day WHO declaring COVID-19 as a global pandemic, marked with a vertical green solid line). Not surprisingly, the time series pattern of the mean and percentiles of the simulated risk scores are quite similar to that of the network density in Fig. 2.

**Fig. 6:**
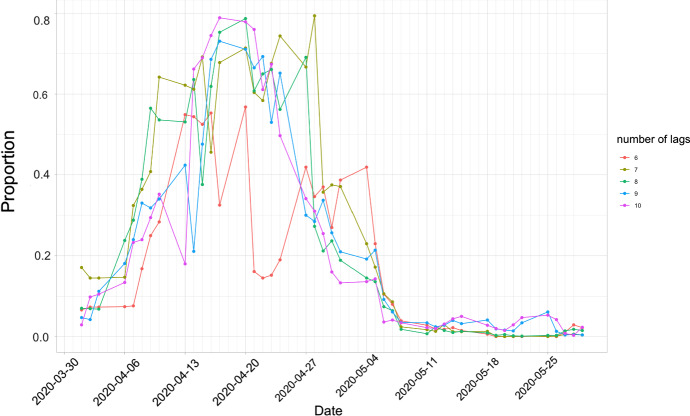
Proportion of significant cases ($\alpha =0.1$) simulated from 1000 cases for each numbers of lag from 6 to 10

To investigate the lead–lag relationship between the pandemic network density and the portfolio risk score $S_{it}$, we perform Granger causality test in (1) by replacing the financial network density with $S_{it}$ in $d_t^{(F)}$, i.e. setting $d_t^{(F)}= \log S_{it} - \log S_{i(t-1)}$. We perform the Granger causality test again using a moving window mechanism with window size of 40 to test if pandemic network density leads the portfolio risk score. We calculate the proportion of significant cases on each day. Figure 6 shows the time series of the proportions of significant cases on each day, with the Granger causality tests using 6 to 10 lags. The results with different $p=q$ are quite consistent. When COVID-19 confirmed cases were widely publicized and the pandemic started to evolve in February 2020, the proportion of random portfolios being significantly Granger-caused by the pandemic network density increased from *t* in early April, which corresponds to the window started in early February, indicating stronger contagion from the pandemic to the financial markets was recorded since February 2020. The proportions of significant cases reached the peaks in mid April. For example, in $p=q=10$, the proportion attained the maximium of around 0.8 on 17 April 2020, and the proportions near 17 April 2020 were also high. In other words, around 80% of the random portfolios were Granger-caused by the pandemic network density in mid April. The pandemic effect on the portfolio risk started to diminish in the second half of April to May 2020. In May 2020, almost no $S_{it}$ was significantly affected by the pandemic network density.

## Discussion

In this paper, we study the financial market connectedness and systemic risk during the COVID-19 pandemic. We observe that financial markets were more connected after the pandemic risk has increased substantially. To further test the lead-lag relationship between the pandemic network connectedness and the financial network connectedness, we conduct Granger causality tests. We find that there is strong evidence that the financial network density was Granger-caused by the pandemic network density from February to April 2020. In the Monte Carlo simulation experiment, we show that the risk of many of random portfolios is Granger caused by the pandemic network density from March to May 2020. The leading effect of the COVID-19 pandemic on the systemic risk in the financial market is quite evident. Further research is warranted to incorporate the pandemic network information for modeling dynamic financial networks, from which better systemic risk assessment can be expected.
